# Seawater immersion aggravates sciatic nerve injury in rats

**DOI:** 10.3892/etm.2015.2281

**Published:** 2015-02-09

**Authors:** HAIFENG WANG, JIAN FANG, FENG HU, GEWEI LI, HE HONG

**Affiliations:** 1 Department of Micro-Orthopedics, The People’s Liberation Army Clinical College Affiliated with Anhui Medical University (The 105th Hospital of People’s Liberation Army), Hefei, Anhui 230031, P.R. China; 2 Department of Orthopedics, The People’s Liberation Army Clinical College Affiliated with Anhui Medical University (The 105th Hospital of People’s Liberation Army), Hefei, Anhui 230031, P.R. China

**Keywords:** seawater immersion, sciatic nerve injury, neuronal recovery, oxidative stress

## Abstract

The aim of the present study was investigate the impact of seawater immersion on peripheral nerve injury and the underlying mechanisms. A total of 234 specific pathogen-free Sprague-Dawley male rats were randomly divided into a sham group, injury control group and seawater immersion + injury group. The Sciatic Functional Index (SFI) was used to assess nerve function for 6 weeks after injury. Compound muscle action potentials were measured and hematoxylin and eosin (H&E) staining of nerve specimens was carried out at week 6. Levels of reactive oxygen species (ROS) and malondialdehyde (MDA) in nerve tissues were measured by enzyme-linked immunosorbent assay (ELISA), and the expression levels of inducible nitric oxide synthase (iNOS) mRNA and protein were measured by reverse transcription-quantitative polymerase chain reaction and immunohistochemistry, respectively. The SFI value in the seawater immersion + injury group after 6 weeks was lower than that in the injury control group (P<0.05). The compound muscle action potential in the seawater immersion + injury group had a prolonged latency, and the amplitude and nerve conduction velocity were decreased compared with those in the other groups (P<0.05). H&E staining demonstrated that nerve fiber regeneration was worse in the seawater immersion + injury group. The ROS and MDA levels in the seawater immersion + injury group were higher than those in the other groups (P<0.05). The expression levels of iNOS mRNA and protein gradually increased in the injury and seawater immersion + injury groups and peaked at 48 h after surgery. Immersion in seawater further aggravated sciatic nerve injury and led to worse neuronal recovery. The mechanism may be associated with oxidative stress.

## Introduction

Peripheral nerve injury often leads to various degrees of permanent dysfunction. Although peripheral nerve injury has been extensively studied, the biochemical, immunological and neurological pathology following nerve injury has not been fully elucidated. The primary factor in peripheral nerve injury is primary axonal injury caused by physical mechanics. The secondary injury is caused by a cascade of inflammatory reactions, including vascular permeability changes and macrophage invasion (1). The internal environment of nerve fibers changes with peripheral nerve injury and is affected by a variety of local factors (including cytokines and humoral factors), which directly or indirectly impact the injury and regeneration reactions of peripheral nerves. Nerve tissues are particularly sensitive to oxidative stress [excessive reactive oxygen species (ROS) and reactive nitrogen radicals], and excessive quantities of free radicals affect functional recovery following nerve injury (2,3). It has been suggested that the expression of nitric oxide synthase (NOS)-1 in the spinal cord might be responsible for the maintenance of chronic peripheral neuropathic pain in mice (4). There is evidence that an increase in the level of inducible NOS (iNOS) expression following sciatic nerve injury in rats may play a role in nerve regeneration and that excessive iNOS expression following nerve injury may hinder nerve regeneration (5). Lipid peroxidation caused by free radicals plays an important role in tissue damage following peripheral nerve injury (6,7). Therefore, diseases associated with oxidative stress, drugs or environmental factors can affect functional recovery after nerve injury (8).

Compared with body fluids, seawater provides a hypertonic, high-sodium and high-alkali environment. With a low temperature, a high osmotic pressure and many kinds of bacteria, seawater entering a body cavity or seawater immersion of a wound may result in more serious problems that need to be urgently addressed during trauma treatment. Few researchers have studied on open injury combined with seawater immersion. There have been studies on dermatoses caused by marine organisms and the impact of seawater drowning injury (9,10), as well as the effects of various rewarming methods on seawater immersion-induced hypothermia (11). Until now, some studies on injuries with seawater immersion have involved superficial soft tissue injury, limbs injury, traumatic brain injury and open chest and abdominal injuries (12–14). Pan *et al* studied topical dorsal skin immersion in seawater in mice and reported that immersion can cause time-dependent apoptosis and proliferation in the epidermis (15). Chen *et al* (16) found in a rabbit model of a firearm-induced limb wound combined with seawater immersion that lipid peroxidation in the firearm injury immersed in seawater was strengthened, which thereby increased peroxidation of the injury (15). In a study of gunshot wounds of rabbits, Liu *et al* found that with concomitant seawater immersion of the femoral arteries there was marked swelling of cells as well as of the intercellular space in the wound tract and area with contusion (17). Nitric oxide (NO) levels in skeletal muscle tissues with firearm injury in limbs immersed in seawater have been found to be significantly higher than those before immersion, which may be associated with NOS activation, leading to secondary injury in skeletal muscle tissues (18).

Although studies have shown that seawater immersion aggravates damage to a variety of human organs and tissues, it remains unknown whether it increases peripheral nerve injury or delays neuronal recovery. Open hip or thigh injuries often cause sciatic nerve injury. Many previous studies of sciatic nerve injury are based on the terrestrial environment, but there are huge differences, both in physical and chemical properties and biological components, compared with those in the marine environment. The nerve crush injury model is used to study the degeneration and regeneration process of nerve fibers following peripheral nerve injury (19,20). In crush injury, in which there is contusion and laceration caused by open injuries, nerve tissues can be directly in contact with seawater and this immersion in seawater may participate in and influence the development of pathophysiological processes following nerve injury. The conditions may be more serious in sciatic nerve injury. In this study, a rat sciatic nerve crush injury model with seawater immersion was established and the impact of seawater immersion was observed on neuronal recovery and pathological changes following sciatic nerve injury. On this basis, changes in the contents of ROS, malondialdehyde (MDA) and iNOS in injured nerve tissues were detected to explore the possible mechanism of secondary sciatic nerve injury caused by seawater immersion and provide an important theoretical basis for further clinical treatment.

## Materials and methods

### Preparation of artificial seawater

Artificial seawater (ASW) was prepared according to Iannacone *et al* (21) [460 mM NaCl, 10 mM KCl, 10 mM CaCl_2_, 22 mM MgCl_2,_ 26 mM MgSO_4_ and 10 mM 2-[4-(2-hydroxyethyl)-1-piperazinyl]ethanesulfonic acid (HEPES; pH 7.8)].

### Animals and grouping

A total of 234 specific pathogen-free Sprague-Dawley (SD) male rats that were 8 weeks old and weighed 200–250 g were provided by the Experimental Animal Center of Anhui Medical University (Hefei, China). The animals were randomly divided into three groups with 78 rats in each group, namely the sham group, injury control group and seawater immersion + injury group. In the injury control group, the sciatic nerve was crushed with an artery clip. In the seawater immersion + injury group, after the sciatic nerve was crushed, rats were immersed in ASW for 1 h followed by wound debridement and suturing. In the sham group, the rats received a surgical incision similar to that in the other two groups but without nerve injury or seawater immersion. Animal feeding and management were performed according to the guidelines for animal experiments at Anhui Medical University. All rats were fed in dedicated cages with 6 rats in each cage. Under consistent feeding and management conditions, the rats were fed dedicated rat food and drank water freely and had 12 h of illumination time per day. All experimental procedures involving animals were approved by the Institutional Animal Care and Use Committee of Anhui Medical University.

### Surgical treatment

Rat sciatic nerve crush injury was performed according to the procedure used by Pan *et al* (22). Rats were intraperitoneally injected with 3% sodium pentobarbital (30 mg/kg) for anesthesia and were fixed in the prone position. An incision was made in the middle of the rear left femoral area and 3 cm sciatic nerve was exposed from the lower edge of the piriformis to above the knee. One centimeter under the piriformis, the sciatic nerve was clamped with an artery clip and was crushed from the opposite direction twice, for 30 sec each time. Microsutures (l0-0) were used to mark the distal injury. In the seawater immersion + injury group, after the sciatic nerve was crushed, the rats were immersed in ASW for 1 h followed by wound debridement and suturing. Among the three groups, two rats died from an overdose of anesthesia. Also, four rats in the seawater immersion group died within 2 h after immersion. The timely replenishment of rats was carried out.

### Tissue preparation and slicing

Sciatic nerve tissues were drawn prior to and at 2, 6, 24 and 48 h, and 1 and 6 weeks after surgery. Rats were anesthetized with 3% sodium pentobarbital and the injured side of the sciatic nerve was exposed, after which 1 cm sciatic nerve was removed from the distal injury and was frozen at −70°C. For each group, six samples were preserved and the other six were fixed in 4% paraformaldehyde followed by dipping in wax, embedding and sectioning (thickness, 4 μm).

### Sciatic nerve function assessment

The Sciatic Functional Index (SFI) was used for the assessment according to Pan *et al* (22). A 50×10-cm walking track was prepared with a piece of white paper of equal length and width placed at the bottom of the track. The rat plantar was dipped into carbon ink and three or four bilateral hind foot prints were clearly recorded. The SFI value of each group was calculated by the Bain formula as follows: SFI = −38.3(EPL-NPL)/NPL + 109.5(ETS-NTS)/NTS + 13.3(EIT-NIT)/NIT − 8.8, where EPL is the experimental print length; NPL is the normal print length; ETS is the experimental toe spread; NTS is the normal toe spread; EIT is the experimental intermediary toe spread; and NIT is the normal intermediary toe spread. The print length, the toe spread and the intermediary toe spread were obtained by measuring the prints of experimental and normal feet. The data were accurate to the millimeter. SFI = 0 corresponds with normal function and 100 with complete dysfunction. All groups were assessed prior to surgery, and at 24 h and each weekend of weeks 1–6 after surgery. At each time-point, six rabbits were randomly selected for each group.

### Electrophysiological testing

Electrophysiological testing was performed using the evoked potential/electromyography measuring system Neuropack M1 MEB-9200K (Nihon Kohden Tomioka Corporation, Tomioka, Japan). Following exposure of the sciatic nerve, two bipolar-protected electrodes were placed 0.5 cm from both the far and near ends of the injured nerve as the stimulating electrodes. The recording electrode was a monopolar center needle electrode obliquely inserted into the middle of the muscle belly in the triceps surae muscle to record the compound muscle action potential and calculate the amplitude, latency and motor nerve conduction velocity. All groups were assessed at 6 weeks after surgery and each time six rabbits were randomly selected for each group.

### Morphological pathology observation

At 6 weeks after surgery, nerve specimens were cut into continuous cross-sections and the specimens were stained using hematoxylin and eosin (H&E) to observe nerve fiber regeneration.

### Detection of ROS and MDA in rat nerve tissues

Cryopreserved rat neural tissues were thawed at room temperature and diluted to 1% homogenate. Following centrifugation, the levels of ROS and MDA in the supernatant were detected using a biotin double-antibody sandwich enzyme-linked immunosorbent assay (ELISA) according to the manufacturer’s instructions (E33106, 10417R; Nanjing Jiancheng Bioengineering Institute, Nanjing, China).

### Detection of iNOS

An immunohistochemistry assay was performed using a two-step kit (cat no. PV-6000, Beijing Zhongshan Jinqiao Biotechnology Co., Ltd., Beijing, China). For each group, a section was selected as the negative control to which no iNOS monoclonal antibody was added. The rabbit anti-rat iNOS polyclonal antibody (cat. no. BS-2072R; 1:200; 9 min incubation at 37°C), biotin-labeled goat anti-rabbit IgG polyclonal antibody (1:300; Beijing Biosynthesis Biotechnology Co., Ltd., Beijing, China; 30 min incubation at 37°C) and horseradish peroxidase-labeled streptavidin working solution (1:300; Beijing Zhongshan Jinqiao Biotechnology Co., Ltd.) 30 min incubation at 37°C and were added sequentially to the sections. Then, diaminobenzidine (DAB) was used for chromogenic reaction (3 min incubation at 20°C) followed by mounting, drying, transparency and mounting with neutral resin. Brown granules were present only in positive cells. Under a 10×20-fold light microscope (BX-43; Olympus Corporation, Tokyo, Japan), 10 non-overlapping fields of each section in the same area were selected for analysis using a multimedia image analysis system (Image-Pro Plus 7.0; Media Cybernetics, Inc., Rockville, MD, USA). The absorbance value was measured, which indicated the relative strength of iNOS expression.

iNOS mRNA expression was detected by reverse transcription-quantitative polymerase chain reaction (RT-qPCR). Total RNA was extracted from cryopreserved sciatic nerves using TRIzol reagent (Thermo Fisher Scientific Inc., Waltham, MA, USA). cDNA was synthesized according to the instructions of the RevertAid™ First Strand cDNA Synthesis kit (Thermo Fisher Scientific Inc.). The design and synthesis of primers used in this study were completed by Life Technologies (Thermo Fisher Scientific Inc.). Rat iNOS primer sequences for fluorescence qPCR were, forward: 5′-GTTCTTTGCTTCTGTGCTAATGC-3′ and reverse: 5′-AGTTGTTCCTCTTCCAAGGTGTT-3′. β-actin was used as an internal reference and its primer sequences were, forward: 5′-CCCATCTATGAGGGTTACGC-3′ and reverse: 5′-TTTAATGTCACGCACGATTTC-3′. qPCR analysis was performed using the SYBR Green PCR kit (Qiagen GmbH, Hilden, Germany) on a real-time PCR instrument (Thermo Scientific™ PikoReal™ Real-Time PCR system; Thermo Fisher Scientific, Waltham, MA, USA). A total of 15 ng cDNA of each sample was analyzed by PCR and the reaction conditions were 95°C for 5 min followed by 40 cycles of 95°C for 10 sec and 60°C for 30 sec. Then, the melting curve was analyzed to ensure the quality of the PCR products. The gene of interest in each tissue was calibrated with its corresponding internal reference. The analysis was repeated three times for each sample and the relative quantitative analysis was performed using the 2^−ΔΔCt^ method.

### Statistical analysis

Data are shown as mean ± standard error and were analyzed using SPSS statistical software, version 13.0 (SPSS, Inc., Chicago, IL, USA). An α-value of P<0.05 was considered statistically significant. Student’s t-tests and one-way analysis of variance were used for pairwise comparison and multiple comparisons, respectively.

## Results

### SFI values

**Figure 1:**
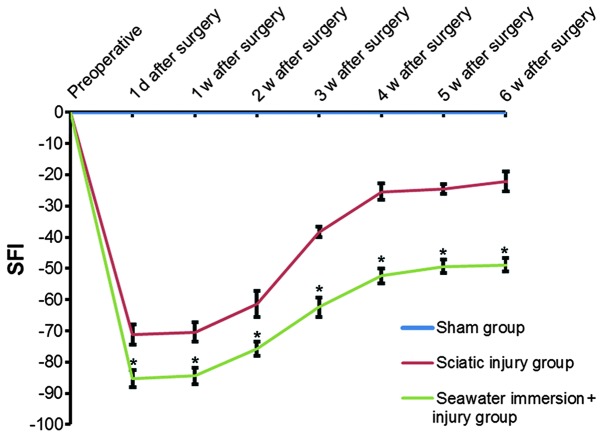
Sciatic Functional Index (SFI) values in the immersion + injury group at 1–6 weeks (w) after surgery were significantly lower than those in the injury group. ^*^P<0.01 compared with the sham and injury groups.

No significant changes were found in the rat feet in the sham group. In the injury and seawater immersion + injury groups, the print length of the injured hind feet was longer and the toe spread and intermediary toe spread were narrower than that in the sham group. On the day following injury, the SFI values were −71.21±3.13 and −85.35±2.78 for the injury and seawater immersion + injury groups, respectively (P<0.05). At one week after injury, rats in the two injury groups went through a slow and gradual recovery process, which increased at 2–4 weeks and tended to be stable at 5–6 weeks. The SFI values were significantly lower in the seawater immersion + injury group compared with those in the injury group (P<0.05, Fig. 1).

### Electrophysiological testing

**Figure 2:**
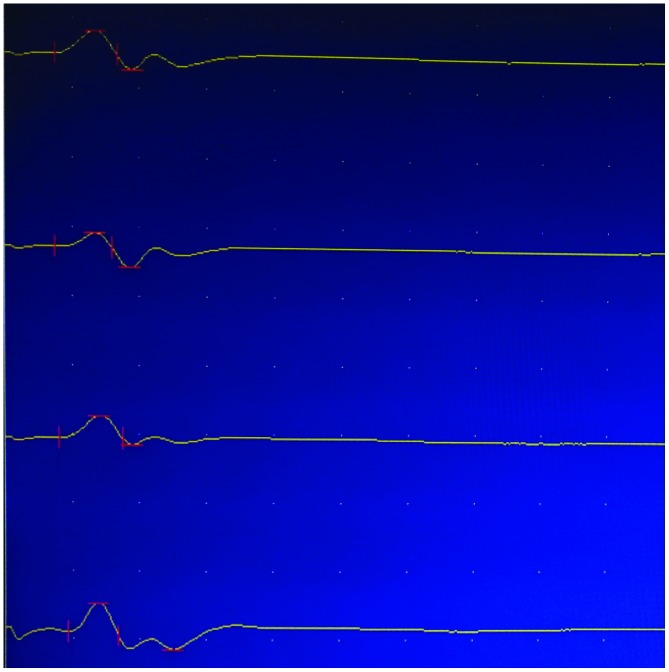
Compound muscle action potential waveforms of the seawater immersion + injury group.

Results of nerve electrophysiological tests carried out at 6 weeks after injury are shown in Table I. Compound muscle action potentials of the seawater immersion + injury group are shown in Fig. 2. The latency was prolonged and the amplitude and nerve conduction velocity decreased in the seawater immersion + injury group compared with those in the sham group. Comparisons between the three groups are shown in Table I. Differences between the groups were statistically significant (P<0.05).

### Pathological results

**Figure 3:**
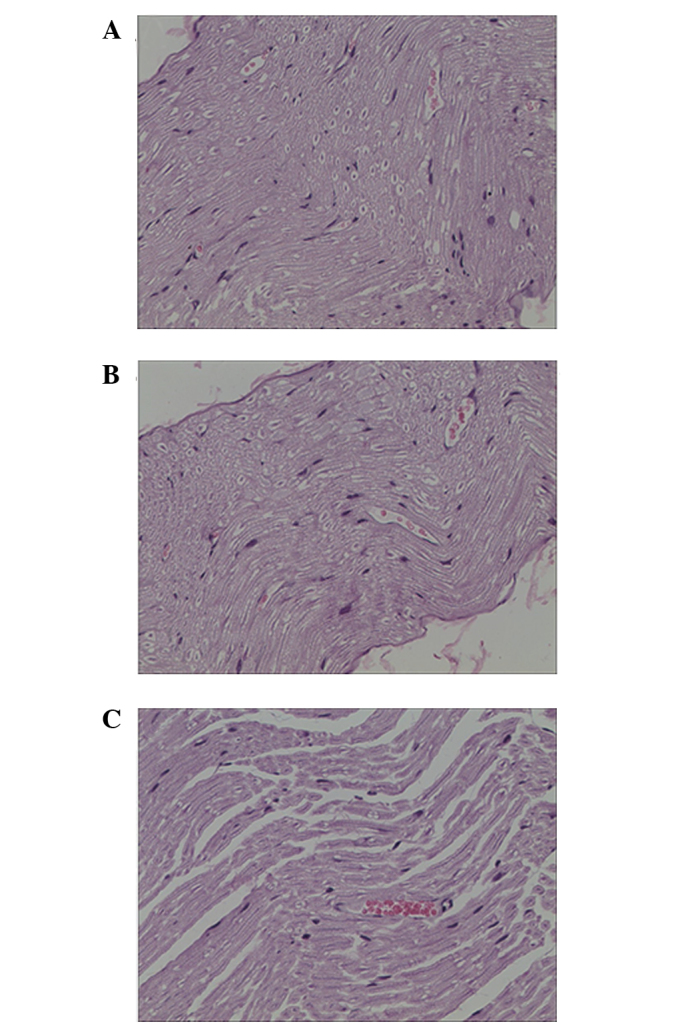
Hematoxylin and eosin (H&E) staining from the three groups 6 weeks after surgery. H&E staining in (A) the sham group, (B) the injury group and (C) the seawater immersion + injury group 6 weeks after surgery (magnification, ×200).

At 6 weeks after surgery, it was observed under the optical microscope in the injury and seawater immersion + injury groups that irregular annular regenerated nerve fibers grew with different thickness, density and number. Following H&E staining, numerous wispy tissues were visible in the injury group and thick nerve fibers were arranged in neat rows. New capillaries were observed between tissues. Wavy wispy tissues were also seen in the seawater immersion + injury group. The nerve fibers were thinner in the seawater immersion + injury group and new capillaries were present between tissues that were mainly collagen tissues (Fig. 3).

### Levels of ROS and MDA in rat nerve tissues

**Figure 4:**
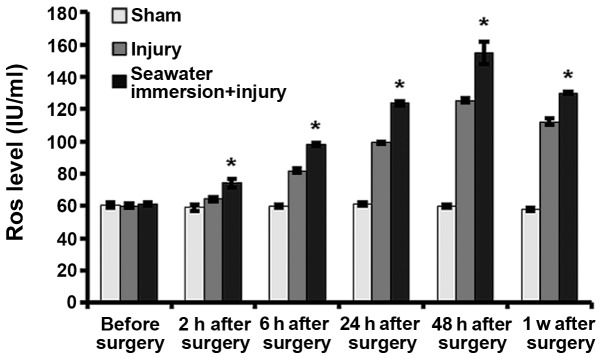
Reactive oxygen species (ROS) levels in the immersion + injury group at 2, 6, 24 and 48 h and 1 week (w) after surgery were significantly higher than those in the sham and injury groups. ^*^P<0.01 compared with the sham and injury groups.

**Figure 5:**
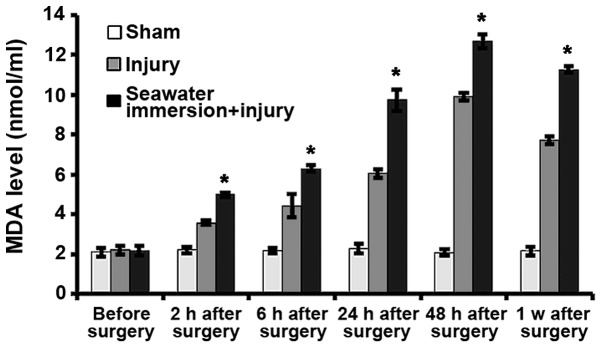
Malondialdehyde (MDA) levels in the immersion + injury group at 2, 6, 24 and 48 h and 1 week (w) after surgery were significantly higher than those in the sham and injury groups. ^*^P<0.01 compared with the sham and injury groups.

It is shown in Figs. 4 and 5 that the level of ROS and MDA in the sham group did not significantly change at each time-point. In the injury and seawater immersion + injury groups, the levels of ROS and MDA gradually increased, peaking at 48 h after injury. The levels of ROS and MDA in the seawater immersion + injury group were higher than those in the sham and injury groups at each time-point.

### Expression of iNOS protein and mRNA in rat sciatic nerve

**Figure 6:**
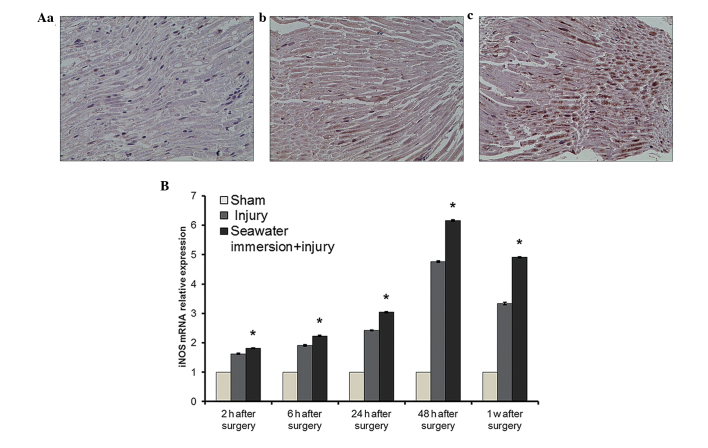
Inducible nitric oxide synthase (iNOS) expression. (A) iNOS expression 48 h after surgery in (a) the sham group, (b) the injury group and (c) the seawater immersion + injury group (magnification, ×200). (B) iNOS expression levels in the immersion + injury group at 2, 6, 24 and 48 h and 1 week (w) after surgery were significantly higher than those in the sham and injury groups. ^*^P<0.01 compared with the sham and injury groups.

Small amounts of iNOS-positive products were detected in the sciatic nerve of the sham group. Following nerve surgery, iNOS positive products gradually increased in quantity in the injury and seawater immersion + injury groups, peaking at 48 h after surgery. iNOS-positive cells were mainly macrophages and Schwann cells with cytoplasm that was brownish yellow or brown with uneven depth (Fig. 6A). The average absorbance (A value) increased gradually and peaked at 2 days after surgery, after which it gradually decreased.

The expression level of iNOS mRNA in the sciatic nerve in the sham group was very low at all time points. In the injury and seawater immersion + injury groups, the level of iNOS mRNA expression gradually increased and peaked at 48 h after surgery. There were significant differences in the levels of iNOS mRNA expression between each time-point in the injury and seawater immersion + injury groups. The difference in mRNA expression level was significant between the injury group and seawater immersion + injury group at each time-point (P<0.01, Fig. 6B).

## Discussion

Sciatic nerve crush injury is a relatively mild and irreversible nerve injury that is frequently used in studies of nerve regeneration (23,24). Motor function, mainly measured by the SFI and Basso, Beattie and Bresnahan scores (22,25), can directly reflect nerve function following peripheral nerve injury. In the present study, severe limb dysfunction in rats was found following both pure sciatic nerve crush injury and sciatic nerve crush injury combined with seawater immersion; however, in the seawater immersion + injury group the dysfunction was more serious. In the first 2–4 weeks after injury, a fast functional recovery was observed, which remained at a relatively stable status at 5–6 weeks. Previous studies have shown that nerve function returns to a stable state 2–4 weeks after sciatic nerve crush injury (19,26), which is consistent with the results of the present study. In this study, the SFI value in the seawater immersion + injury group was lower than that in the injury group and the difference was statistically significant, suggesting that seawater immersion aggravated sciatic nerve injury and hindered the recovery of neurological motor function.

The main function of peripheral nerves is to conduct nerve impulses to the spinal cord and brain to form somatic or visceral sensory sensations and conduct nerve impulses from the brain and spinal cord to enable somatic or visceral movement. Neuronal recovery is the key for determining the effects of nerve regeneration after peripheral nerve injury. Nerve conduction, the basic function of peripheral nerves, can be directly reflected by the conduction velocity of the electrical activity of the neural stem. The amplitude of the compound muscle action potential is proportional to the number and size of regenerated axons (27). Nerve conduction velocity is associated with the thickness and maturity of the myelin sheath. A fast nerve conduction velocity can indirectly indicate a thick and matured myelin sheath of regenerated nerve fibers (28). In the present study, it was shown in Table I that the amplitude and nerve conduction velocity of rat sciatic nerve action potentials in the seawater immersion + injury group were lower than those of the sham and injury groups (P<0.05), suggesting that seawater immersion hindered axon regeneration and that the thickness and maturity of the myelin sheath were less than that of the control groups. These results indicate that seawater immersion weakened the recovery of nerve conduction following sciatic nerve injury.

The response to and regeneration of the peripheral nervous system following injury varies with the cause and extent of injury. Pathological changes following peripheral nerve injury also depend on the degree of injury. Wallerian degeneration following peripheral nerve injury consists of a series of processes, including axonal degeneration, myelin degeneration and disintegration, Schwann cell proliferation, infiltration of macrophages and mast cells, and axonal and myelin debris clearance (29). Nerve fiber degeneration after peripheral nerve injury is the response to injury and the process of preparing for nerve regeneration. Peripheral nerve regeneration is the process that occurs after the fracture of peripheral nerve axons, when degenerated axons and myelin are cleared with the establishment of a regenerated micro-environment, after which nascent buds grow from axons proximal to the injury and extend along the regenerative channel to target organs and contact with them to achieve reinnervation of target organs (30). Histology is a traditional method to evaluate the recovery of regenerated nerves. Nerve regeneration can be indirectly reflected by histomorphological parameters, including myelinated nerve fibers, regenerated axon diameter and myelin sheath thickness. In the present study, histological examination of regenerated nerves was conducted by conventional staining. At 6 weeks after surgery, relatively larger number of nerve fibers were observed in the injury group, as well as axons with a long diameter, whereas few nerve fibers were visible in the seawater immersion + injury group. The pathological findings were consistent with the neural electrophysiological results, indicating that nerve regeneration in the injury group was better than that in the seawater immersion + injury group, which strongly suggests that seawater immersion inhibited peripheral nerve regeneration.

Hyperosmosis, high alkalinity, low temperature and bacteria are generally accepted injury factors. Compared with body fluids (including intracellular fluid and extracellular fluid), seawater provides an environment of high sodium levels, hyperosmosis and high alkalinity. Shapiro and Dinarello (31) demonstrated that peripheral blood mononuclear cells expressed interleukin-8 (IL-8) mRNA and synthesized IL-8 in hypertonic fluid. The synergistic effect of IL-8 with bacterial lipopolysaccharide increased the synthesis of tumor necrosis factor (TNF), and this increased as the acting time of the hypertonic stimulating factor was prolonged. TNF can directly activate monocytes/macrophages and neutrophils, resulting in increased superoxide anion levels and tissue injury. Local exposure of injured tissues to hypertonic seawater will lead to intracellular dehydration and edema between tissues, which in turn can easily lead to changes in intracellular and extracellular ion concentrations and increase the burden on the cell membrane ion pump (32). Furthermore, the low temperature of seawater causes depletion of adenosine triphosphate, resulting in a more active metabolism with significantly increased requirements of oxygen and glucose, after which the reduction products are increased in quantity, inducing a redox reaction. Subalkaline seawater causes an imbalance of intracellular and extracellular ion concentrations and increases the cell response to injury (33). Under subalkaline conditions, damaged cells are more likely to disintegrate, leading to the release of membrane phospholipids and to lipid peroxidation (34). Furthermore, tissue damage may result in the saponification of fats or soluble basic protein, which further increases the damage to the tissues (35). However, until now, investigations concerning the mechanism by which seawater immersion affects open injury have been lacking.

Peripheral nerves undergo a process of nerve ischemia and inflammation after injury, during which oxygen radicals and many toxic substances gather at the injury site, changing the membrane permeability and stimulating calcium influx. The proteolytic pathway is then activated, which leads to cell damage, including damage to neurofilaments and microtubules (36). Sayan *et al* (37) reported similar findings in a sciatic nerve ischemia-reperfusion injury model, and hypothesized that this may be attributable to the myelin being rich with lipids, which are the main target in the free radical-mediated lipid peroxidation process. Liao *et al* (38) found that after the sciatic nerve is crushed or tied, large amounts of oxygen free radicals are produced, which may have an impact on peripheral nerve regeneration. Oxygen radicals not only damage phospholipids in nerve membranes, but also make myelin protein more vulnerable to attack by ROS. Oxygen free radical-induced lipid peroxidation is an important factor in the degeneration of nerve tissue following injury. MDA is a lipid product formed by the action of free radicals on unsaturated fatty acids in the cytoplasm, where two or more double bonds of the unsaturated fatty acid are fractured after being damaged by oxygen free radicals in the body. The determination of MDA content can reflect the degree of lipid peroxidation in the body and indirectly reflect the severity of the attack on cells by free radicals (39). The present study demonstrated that the levels of ROS and MDA in nerve tissues were increased following sciatic nerve injury, and the levels of ROS and MDA were higher in the seawater immersion + injury group than those in the sham and injury groups. Also, the levels of ROS and MDA peaked at 48 h after injury and were maintained at a high level for one week, indicating that seawater immersion aggravated oxidative stress of nerve tissues, lipid oxidation and nerve injury.

Reactive nitrogen comes from nitric oxide (NO), and NOS is a major rate-limiting enzyme in the synthesis of NO in the body. NOS includes neuronal NOS, endothelial NOS and iNOS. iNOS mainly exists in macrophages and neutrophils and can be activated in a variety of ways following tissue damage when excess NO is produced. After nerve injury, iNOS is highly expressed and excessive NO is produced, causing cell toxicity and further damage to the nerve, which is not conducive to nerve regeneration. Shin *et al* (40) reported that iNOS expression was significantly increased following nerve ischemia and reperfusion, and that iNOS synthesis was inhibited by an iNOS specific inhibitor; this reduced NO production and thus may play a protective role in peripheral nerve ischemia-reperfusion injury. In the present study, the expression of iNOS protein and mRNA was detected. The results revealed that the content of iNOS in nerve tissues was increased after sciatic nerve injury, and was higher in the seawater immersion + injury group than in the sham and injury groups, peaked at 48 h after injury and was maintained at a high level for one week. This indicated that seawater immersion stimulated iNOS expression and increased reactive nitrogen, thereby aggravating the oxidative stress to nerve tissues and increasing nerve injury.

There are few studies that have investigated the effect of seawater immersion on peripheral nerve injury. The present study demonstrated that seawater immersion aggravated nerve injury, hampered the recovery of neurological function, and increased the oxidative stress in nerve tissues, as indicated by increased ROS and MDA production and high expression levels of iNOS. Further studies will be carried out to elucidate how oxidative stress mediates the aggravating role of seawater immersion in sciatic nerve injury.

## Figures and Tables

**Table I:** Comparison of electrophysiological test results 6 weeks after injury.

Groups	n	Latency (msec)	Amplitude (mV)	Conduction velocity (m/sec)
Sham	6	2.13±0.24	33.21±1.59	60.45±3.29
Injury	6	4.21±0.75	26.10±1.47	20.71±2.67
Seawater immersion + injury	6	4.86±0.43^a^	3.62±1.12^a^	16.45±2.35^a^

a P<0.01 compared with the sham and injury groups.
